# First-Principles Study of Topological Nodal Line Semimetal I229-Ge_48_ via Cluster Assembly

**DOI:** 10.3390/nano15141109

**Published:** 2025-07-17

**Authors:** Liwei Liu, Xin Wang, Nan Wang, Yaru Chen, Shumin Wang, Caizhi Hua, Tielei Song, Zhifeng Liu, Xin Cui

**Affiliations:** Inner Mongolia Key Laboratory of Microscale Physics and Atom Innovation, School of Physical Science and Technology, Inner Mongolia University, Hohhot 010021, China; l18648294612@126.com (L.L.); 15847608829@163.com (X.W.); 13296920141@163.com (N.W.); 17684876370@163.com (Y.C.); 13934746601@163.com (S.W.); 18847529034@163.com (C.H.); tlsong@imu.edu.cn (T.S.); zfliu@imu.edu.cn (Z.L.)

**Keywords:** germanium, mechanical anisotropy, nodal line semimetals, optical properties

## Abstract

Group IV element-based topological semimetals (TSMs) are pivotal for next-generation quantum devices due to their ultra-high carrier mobility and low-energy consumption. However, germanium (Ge)-based TSMs remain underexplored despite their compatibility with existing semiconductor technologies. Here, we propose a novel I229-Ge_48_ allotrope constructed via bottom-up cluster assembly that exhibits a unique porous spherical Fermi surface and strain-tunable topological robustness. First-principles calculations reveal that I229-Ge_48_ is a topological nodal line semimetal with exceptional mechanical anisotropy (Young’s modulus ratio: 2.27) and ductility (*B*/*G* = 2.21, *ν* = 0.30). Remarkably, the topological property persists under spin-orbit coupling (SOC) and tensile strain, while compressive strain induces a semiconductor transition (bandgap: 0.29 eV). Furthermore, I229-Ge_48_ demonstrates strong visible-light absorption (10^5^ cm^−1^) and a strong strain-modulated infrared response, surpassing conventional Ge allotropes. These findings establish I229-Ge_48_ as a multifunctional platform for strain-engineered nanoelectronics and optoelectronic devices.

## 1. Introduction

Since the successful synthesis of Cd_3_As_2_ and Na_3_Bi [1,2] in experiments, topological semimetals (TSMs) have revolutionized condensed matter physics through their exotic electronic states, such as Dirac/Weyl fermions and symmetry-protected nodal lines [3,4,5]. This prominence is attributed to their exceptional properties, including highly efficient catalysis [6,7], low-energy quantum transport [1,8], and negative magnetoresistance [9,10]. These characteristics collectively provide a robust foundation for developing the next generation of low-power and ultra-high-speed electronic devices. A defining feature of TSMs is the presence of band crossings and linear dispersion near the Fermi level in their electron band structure [11]. Based on critical attributes of the band crossing, such as the degree of simplicity, cosine dimension number, and band dispersion, TSMs can be categorized into Dirac semimetals (DSMs) [12,13,14], Weyl semimetals (WSMs) [15,16,17], and topological nodal line semimetals (TNLSMs) [18,19,20]. Each category exhibits unique properties that make them suitable for different applications within advanced electronic and quantum technologies.

The distinctive electronic configuration of group IV elements characterized by an *s*^2^*p*^2^ orbital arrangement allows for *sp*-, *sp*^2^-, and *sp*^3^-hybridized modes, enabling the formation of diverse crystal structures with varying physical properties. Among group IV elements, graphene and silicene dominate TSM research [21,22,23], and a series of three-dimensional (3D) TSMs has been developed based on the 2D graphene structure through material design and assembly. Examples include the interpenetrated graphene network (IGN) [24], triangle graphene network (TNG) [25], carbon-Kagome-lattice (CKL) family [26], and interpenetrating silicene networks (ISN) [27]. Germanium (Ge) remains a promising, yet underexplored, candidate. Ge offers intrinsic advantages, including high carrier mobility comparable to silicon [28,29], low power consumption [30], and diverse allotropes (e.g., Ge-I, Ge-II, Ge-III) [31,32,33] with tunable electronic properties. However, existing Ge-based TSMs suffer from limited topological protection or poor stability [34], highlighting the need for novel structural designs.

Traditional Ge allotropes, such as diamond-cubic Ge-I, metallic Ge-II, and the Ge_12_ cluster [35], exhibit limited topological features. Recent theoretical efforts, however, have unveiled exotic Ge-based phases like Dirac semimetal (germancite, Ge_1−x_Sn_x_) [12,36], nodal line semimetal (ABW-Ge_4_, Ba_2_Ge) [37,38], and topological insulators (germanene, α-Sn_1−x_Ge_x_) [39,40]. These discoveries underscore the potential of Ge allotropes in topological physics but also highlight a critical gap: the lack of stable, low-density Ge structures with tunable properties. Cluster assembly provides a bottom-up strategy to engineer materials with tailored properties [41]. Recent advances in carbon/silicon TSMs demonstrate the potential of this approach. Yet, analogous Ge-based architectures remain scarce.

Here, we propose a novel Ge allotrope, I229-Ge_48_, constructed via bottom-up assembly of germanium clusters. Using first-principles calculations, we demonstrate that I229-Ge_48_ is a topological nodal line semimetal with unique mechanical anisotropy, strain-responsive electronic states, and exceptional optical absorption. This work expands the family of Ge-based TSMs and pave the way for significant advancements in low-energy quantum transport and nanoelectronic devices.

## 2. Computational Methods

First-principles calculations are performed using the Vienna Ab initio Simulation Package (VASP) with projector-augmented wave (PAW) pseudopotentials [42,43,44]. Electronic exchange and correlation effects are treated with the generalized gradient approximation (GGA) functional, specifically the Perdew–Burke–Ernzerhof (PBE) form [45,46,47]. A plane-wave basis set with a kinetic energy cutoff of 400 eV ensures that structural optimization converges to less than 0.001 eV/atom. The Brillouin zone is sampled using a *k*-point grid with a density of 2π × 0.03 Å^−1^, employing the Gamma Scheme method. Convergence criteria for energy and force constant are set to 1 × 10^−6^ eV and 0.01 eV/Å, respectively. Phonon frequencies are calculated using the Density Function Perturbation Theory (DFPT) method, as implemented in the PHONOPY package [48]. To explore finite temperature effects and dynamic behavior, molecular dynamics simulations with a Nosé–Hoover thermostat are employed to evaluate the thermal stability of a 2 × 2 × 1 supercell containing 96 atoms at 500 K within 5 ps [49]. The IRVSP_V1 [50] software package is utilized to calculate and analyze irreducible representations near band degeneracies or nodes, aiding in the identification potential band inversions. A tight-binding model is constructed based on Wannier90 [51], and its Fermi surface is calculated in conjunction with the WannierTools-2.6.1 software package [52].

## 3. Results

### 3.1. Geometric Features

**Figure 1:**
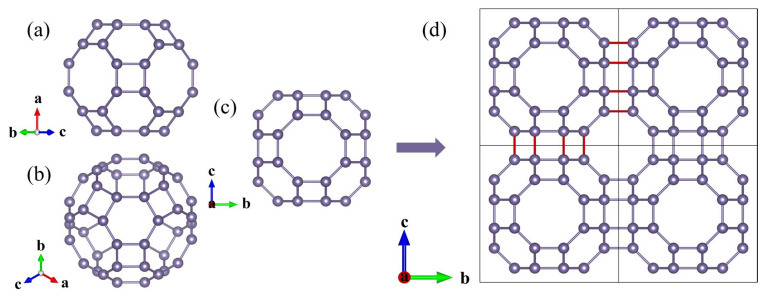
Views of a I229-Ge_48_ nanocage through the normal directions of (**a**) four-, (**b**) six-, and (**c**) eight-membered rings, respectively. (**d**) A 2 × 2 × 1 I229-Ge_48_ supercell.

The I229-Ge_48_ cluster assembly structure (space group *Im*-3*m*, No. 229) forms a nanoporous cubic lattice composed of interconnected 12 four-membered rings, 8 six-membered rings, and 6 eight-membered rings (Figure 1a–c). The selection of this particular nanocage configuration was primarily guided by the precedent set by the successfully synthesized B_24_N_24_ nanocage [53]. The individual cage structures are interconnected by face-to-face bonding of the eight-membered rings, resulting in the formation of eight new Ge-Ge bonds, which are highlighted by red lines in Figure 1d. Each unit cell contains 48 Ge atoms at Wyckoff’s position 48i (0.896, 0.250, 0.396), with equivalent lattice parameters *a* = 12.205 Å along all axes. The structure exhibits three distinct bond lengths (2.517 Å, 2.532 Å, and 2.517 Å) and a low density (3.201 g/cm^3^), attributed to its cage-like porosity. These findings, along with those for other Ge allotropes such as diamond, Ge_12_, oC24, Ge_20_, ST12, and hcp phase, are detailed in Table S1 [54,55]. This nanoporous characteristic of the I229-Ge_48_ crystal structure renders it highly advantageous for applications in heterogeneous catalysis and molecular transport.

### 3.2. Stabilities

**Figure 2:**
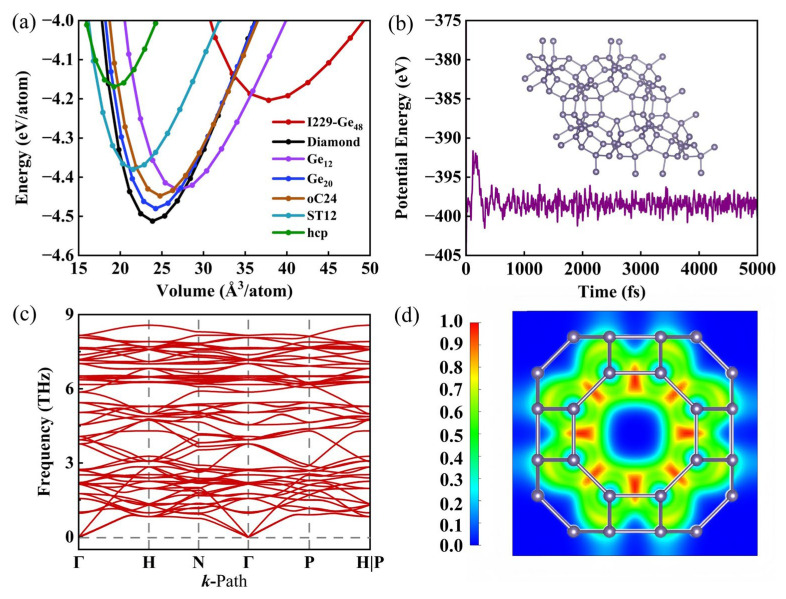
(**a**) Total energy as a function of volume per atom for I229-Ge_48_, together with diamond, Ge_12_, oC24, Ge_20_, ST12, and hcp phase. (**b**) Potential energy fluctuation during 5000 fs FPMD simulations for I229-Ge_48_; the inset is a snapshot of the supercell of I229-Ge_48_ at the end of the simulation. (**c**) Phonon band structure of I229-Ge_48_. (**d**) 2D contour plot of the electron localization function (ELF) of an eight-membered ring.

To assess the stability of the I229-Ge_48_ structure, detailed analysis of the total energy per atom as a function of volume was conducted. This analysis is extended to include a comparative study with six other germanium allotropes––diamond, Ge_12_, oC24, Ge_20_, ST12, and hcp phase––as shown in Figure 2a. Generally, phases with lower equilibrium energy are considered more stable. The third-order Birch–Murnaghan equation of state was employed for this purpose [56]:

(1) $$E_{t}\left(V\right)=E_{0}+\frac{9}{16}B_{0}V_{0}\left[\left(B^{\prime }-4\right){\left(\frac{V_{0}}{V}\right)}^{\frac{2}{3}}-B^{\prime }+6\right]{\left[{\left(\frac{V_{0}}{V}\right)}^{\frac{2}{3}}-1\right]}^{2},$$

where *E*_0_ and *V*_0_ are the energy and volume of the structure at equilibrium, respectively. *B*_0_ is the bulk modulus of elasticity. *B*’ denotes the first derivative of the bulk modulus with respect to pressure. By fitting this equation, we can confirm the stability of the energy. According to the calculation results in Table S1 of the Supplementary Material, the energy difference between I229-Ge_48_ structure and diamond structure is 0.312 eV/atom, which is smaller than the energy difference of 0.339 eV/atom between hcp structure and diamond structure. This indicates that I229-Ge_48_ structure is energetically more favorable than hcp structure, and thus feasible for synthesis.

Assessing the thermal stability of materials is essential for their experimental synthesis and potential practical applications. The results are depicted in Figure 2b, which illustrates the variation of potential energy in relation to the simulation time at 500 K. Throughout the simulation, the I229-Ge_48_ structure maintains its geometry without reconstruction, and its potential energy remains relatively stable within the 0–5 ps range. This consistency indicates that the I229-Ge_48_ structure can endure temperatures up to 500 K without significant structural alterations, thereby demonstrating its thermal stability above room temperature.

To further investigate the dynamical stability of the I229-Ge_48_ structure, we calculate the phonon spectrum, as shown in Figure 2c. The absence of imaginary phonon frequencies across the entire BZ below 0 THz confirms the dynamic stability of the I229-Ge_48_ structure. Moreover, the electron localization function (ELF), a valuable tool for delineating chemical bonds and the extent of electron delocalization in molecules and solids [57], is illustrated for the I229-Ge_48_ structure along the eight ring direction in Figure 2d. The ELF analysis reveals that the Ge-Ge bonds display strong covalent bond characteristics, indicating that the structure possesses excellent chemical stability.

### 3.3. Mechanical Properties

I229-Ge_48_ emerges as a stable cage-like porous germanium allotrope with unique mechanical properties that are crucial for its potential applications under extreme conditions. Elastic constants *C*_ij_ and moduli (bulk modulus *B*, shear modulus *G*) were calculated using the Voigt–Reuss–Hill method. For a comprehensive analysis, we also tabulate the results for six other allotropes (diamond, Ge_12_, oC24, Ge_20_, ST12, and hcp phase) of Ge, as shown in Table 1. The bulk modulus *B* is the arithmetic mean of *B*_V_ and *B*_R_, and *G* equates to the arithmetic mean of *G*_V_ and *G*_R_. The values for *B*_V_, *B*_R_, *G*_V_, and *G*_R_ are determined as follows [58]:

9*B_V_* = (*C*_11_ + *C*_22_ + *C*_33_) + 2 (*C*_12_ + *C*_23_ + *C*_31_), (2)

15*G_V_* = (*C*_11_ + *C*_22_ + *C*_33_) − (*C*_12_ + *C*_23_ + *C*_31_) + 3 (*C*_44_ + *C*_55_ + *C*_66_), (3)

1/*B_R_* = (*S*_11_ + *S*_22_ + *S*_33_) + 2 (*S*_12_ + *S*_23_ + *S*_31_), (4)

15/*G_R_* = 4 (*S*_11_ + *S*_22_ + *S*_33_) − 4 (*S*_12_ + *S*_23_ + *S*_31_) + 3 (*S*_44_ + *S*_55_ + *S*_66_). (5)

Here, *S*_ij_ are the components of the inverse of the compliance matrix *C*_ij_, and for a cubic system, *C*_11_ = *C*_22_ = *C*_33_, *C*_12_ = *C*_23_ = *C*_31_, and *C*_44_ = *C*_55_ = *C*_66_.

The calculate bulk modulus (*B*) and shear modulus (*G*) for I229-Ge_48_ are 31 and 14 GPa, respectively. The Young’s modulus is defined as *Y* = 9*BG/*(3*B* + *G*), and the greater the Young’s modulus (*Y*), the stiffer the material is. According to the calculation results in Table 1, the Young’s modulus is 37 GPa. The *B*/*G* ratio and Poisson’s ratio (*ν* = (3*B* − 2*G*)/[2(3*B* + *G*)]) are critical indicators of a material’s ductility and brittleness [58]. A *B*/*G* ratio below 1.75 and Poisson’s ratio below 0.26 suggest brittleness, while values above these thresholds indicate ductility [64]. Based on the calculated values presented in Table 1, I229-Ge_48_ exhibits a *B*/*G* ratio of 2.21 and a Poisson’s ratio of 0.30, both of which indicate ductile behavior. Compared to other brittle Ge allotropes listed in Table 1, the porous cage-like structure of I229-Ge_48_ demonstrates distinctly different mechanical behavior, potentially making it suitable for applications requiring specific ductile material properties.

**Figure 3:**
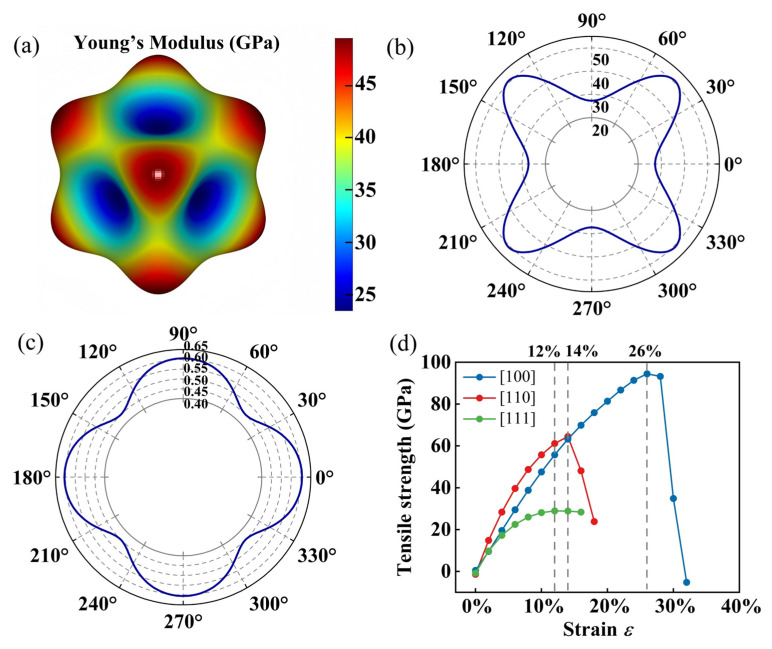
(**a**) Surface contours of Young’s modulus for I229-Ge_48_ in different directions. (**b**) Projected Young’s modulus and (**c**) Poisson’s ratio on the (100) plane. (**d**) Calculated ideal tensile strengths of I229-Ge_48_ along the [100], [110], and [111] directions.

To further investigate the mechanical anisotropy of I229-Ge_48_, we calculate its direction-dependent Young’s modulus using the following formula [65]:

(6) $$Y\left(\theta ,\varphi \right)=\frac{1}{S_{11}-2\left(S_{11}-S_{12}\frac{S_{44}}{2}\right)\left(l_{1}^{2}l_{2}^{2}+l_{2}^{2}l_{3}^{2}+l_{1}^{2}l_{3}^{2}\right)},$$

where $l_{1}=\sin\theta \cos\varphi ,$$l_{2}=\sin\theta \sin\varphi$ and $l_{3}=\cos\theta$ refer to the direction parameters of the three direction axes. The spatial distribution of Young’s moduli across various orientations is meticulously depicted in Figure 3a. From the entire 3D shape and color bar, we can see that the Young’s moduli of I229-Ge_48_ exhibit significant anisotropy, with a ratio of 2.27 between the maximum value (50 GPa) and the minimum value (22 GPa). This anisotropy is evident in the spatial distribution of Young’s moduli and Poisson’s ratios, as depicted in Figure 3b,c. The mechanical anisotropy is further reflected in the tensile strength results, where I229-Ge_48_ exhibits different tensile strengths along the [100], [110], and [111] directions (as shown in Figure 3d). The results indicate that the material can endure tensile strains of 26%, 14%, and 12% prior to yielding, with corresponding tensile strengths of 94.47, 64.49, and 28.93 GPa, respectively. These findings underscore the significant directional dependence in the mechanical properties of I229-Ge_48_.

Finally, to quantify the elastic anisotropy, we calculate the elastic anisotropy index for symmetric crystals using the following formula: *A*^U^ = 5*G*_V_*/G*_R_ + *B*_V_*/B*_R_ − 6 [66]. An *A*^U^ value of zero indicates isotropic behavior, while higher values suggest increased anisotropy. For I229-Ge_48_, we obtain *B*_V_ = *B*_R_ = 31 GPa, *G*_V_ = 15 GPa, a d *G*_R_ = 13 GPa, resulting in an *A*^U^ value of 0.770. This value is notably higher than most germanium allotropes, confirming the significant anisotropy of I229-Ge_48_. In conclusion, I229-Ge_4*8′*_s ductile properties and pronounced mechanical anisotropy, as evidenced by its *B*/*G* ratio, Poisson’s ratio, Young’s modulus, and elastic anisotropy index, distinguish it from other germanium allotropes and suggest its potential for applications where specific mechanical properties are required.

### 3.4. Topological Electronic Properties

**Figure 4:**
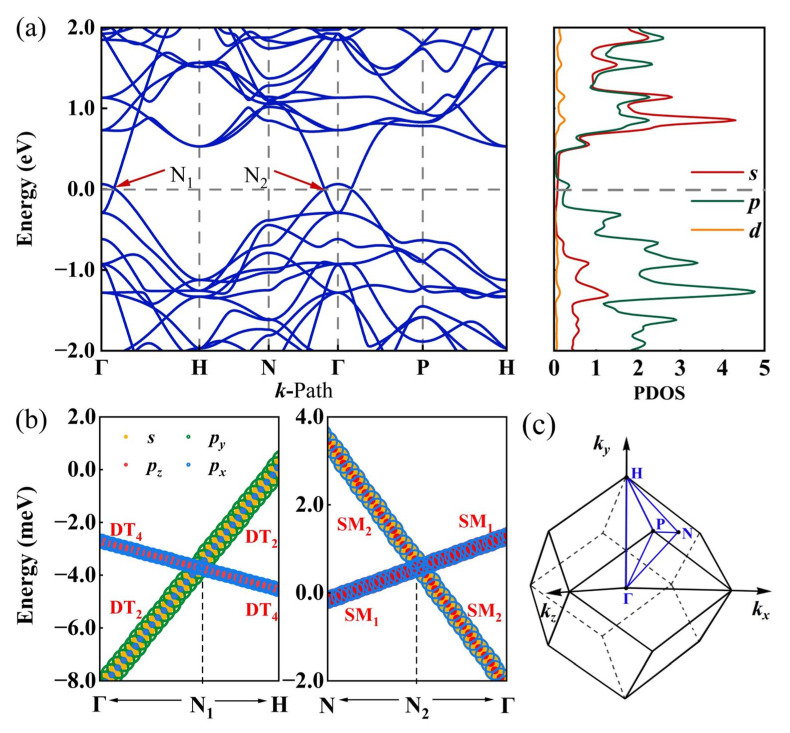
(**a**) The energy band structures of I229-Ge_48_ calculated by PBE theory and the corresponding density of states. (**b**) Orbital projections near the N_1_ and N_2_ nodes without SOC action. (**c**) Schematic representation of highly symmetric points in the BZ.

The energy band structure and density of state (DOS) for I229-Ge_48_ have been determined using the GGA-PBE method. As illustrated in Figure 4a, the results reveal two linear intersections between the valence and conduction bands near the Fermi level along the high symmetry paths Γ-H and N-Γ, designated as N_1_ and N_2_, respectively. Given that the GGA-PBE method is prone to underestimating bandgaps, we employed a more precise approach based on the modified Becke–Johnson exchange potential [67] to ascertain the band structure. As depicted in Figure S1 of the Supplementary Material, the band structure obtained via this refined approach aligns closely with the outcomes of the PBE calculations, preserving the characteristic feature of two distinct nodal points. These intersections are indicative of the material’s electronic properties and are crucial for understanding its behavior as a topological semimetal. In topological semimetals, band crossings near the Fermi level often result from band inversions. To this end, we have calculated the orbital projection, as presented in Figure 4b. Orbital projections indicate band inversion at these nodes, driven by *s*-*p* orbital hybridization. Before the N_1_ point, the energies of the *s* and *p*_y_ orbitals are lower than those of *p*_z_ and *p*_x_ orbitals. However, upon crossing the N_1_ point, there is a significant inversion in the orbital energy levels, leading to a notable change in the energy bands. A similar orbital feature is observed around the N_2_ point, indicating that both intersections result from band inversion. Moreover, the slopes of the two energy bands at these intersections are opposite, classifying these points as type-Ⅰ cone points [68]. Figure 4c illustrates the distribution of each high symmetry point in the BZ, facilitating determination of the plane where the high symmetry paths are located.

**Figure 5:**
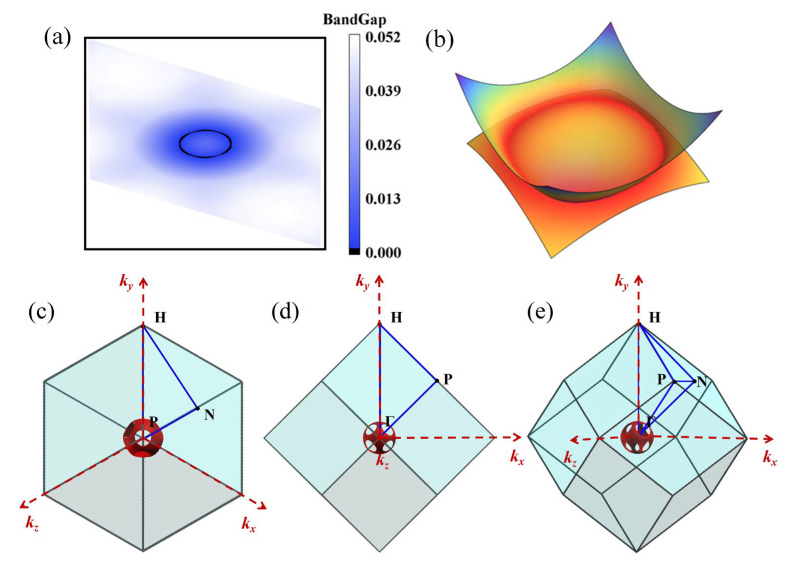
(**a**) Energy bandgap and (**b**) three-dimensional energy band structures along the *k*_x_-*k*_y_ plane. (**c**–**e**) Fermi surface of I229-Ge_48_ in the BZ observed along different orientations.

To ascertain the topological categorization of I229-Ge_48_, we conduct a computation of its band structure along the *k*_x_-*k*_y_ plane within ***k***-space (notably, this plane coincides with the high-symmetry Γ-N-H points) and extend our analysis to its three-dimensional energy band, as illustrated in Figure 5a,b. Both results confirm the existence of a nodal ring in I229-Ge_48_. Nonetheless, considering the material’s exceptionally high symmetry, we hypothesize that its electronic structure might harbor more intricate nodal characteristics, except for a solitary nodal ring.

Based on these results, we further calculate the bandgaps and three-dimensional energy bands along the *k*_x_-*k*_z_ and *k*_y_-*k*_z_ planes, and it can be found that the result is the same with the *k*_x_-*k*_y_ plane. Remarkably, the nodal ring features in these orthogonal planes display identical symmetry-protected characteristics, suggesting that I229-Ge_48_ does not host a single nodal ring. Instead, it is likely a multifaceted topological semimetal with concentrically nested nodal rings, nodal planes, or even a spherical structure. To further elucidate its topological nature, we analyze the Fermi surface. As illustrated in Figure 5c–e, the Fermi surface manifests as a porous sphere within the BZ. This structure displays a hollow feature along the high-symmetry Γ-P path, which is consistent with the presence of bandgap opening in the two-dimensional energy bands along the same path. These findings collectively indicate that I229-Ge_48_ is an unconventional nodal line semimetal. The spherical symmetry of the Fermi surface suggests that it may possess novel properties, such as symmetry-protected surface states or exotic quantum transport behavior.

**Figure 6:**
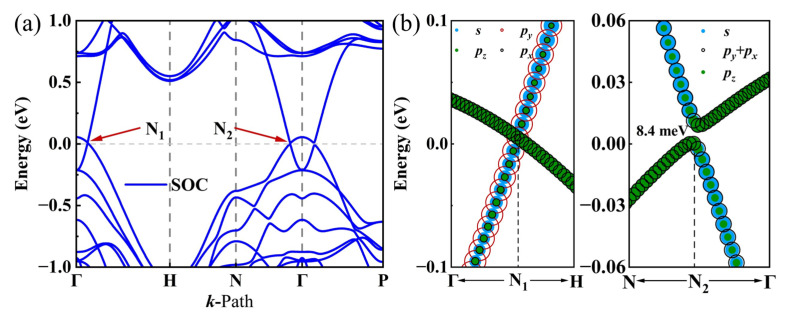
(**a**) Energy band structure and (**b**) orbital projections of N_1_ and N_2_ nodes near the Fermi level with SOC action.

Considering that Ge is a heavy element with *d* orbitals, the incorporation of SOC is imperative. To investigate whether the topological properties persist after including SOC, an orbital analysis is carried out to observe potential band inversion phenomena. As observed in Figure 6a,b, node N_1_ consistently remains gapless, whereas node N_2_ exhibits a minute bandgap of 8.4 meV. Given that the threshold for direct electron leaps typically stands at 26 meV, this marginal bandgap in the I229-Ge_48_ structure can be disregarded under ambient conditions, suggesting that both nodes N_1_ and N_2_ maintain their stability when subjected to SOC effects.

Furthermore, we calculated the Fermi surface of I229-Ge_48_ with SOC included. As shown in Figure S2 of the Supplementary Materials, the opening of the band gap at the N_2_ point modifies the Fermi surface, potentially giving rise to quantum oscillations [69]. Moreover, with the continuous improvement of the Angle Resolved Photoemission Spectroscopy (ARPES) technology, these different Fermi surfaces may be experimentally detected [70,71]. Finally, in order to test whether the nodes of I229-Ge_48_ retain their topological properties when disturbed, we explored its symmetry under SOC. Through computation of the ℤ_2_ topological invariant, it was revealed that I229-Ge_48_ constitutes a weakly topological material with a ℤ_2_ index of (0; 001) [72]. This finding signifies that the impact of SOC on this system is minimal, thereby affirming preservation of its topological properties even when subjected to disturbances.

In comparison to the aforementioned topological nodal line semimetal ABW-Ge_4_, I229-Ge_48_ exhibits a suite of distinctive features. Despite both materials being germanium allotropes and sharing nanoporous structures, the cage-like porous structure of I229-Ge_48_ confers a significant advantage in molecular transfer and storage over ABW-Ge_4_. Moreover, their physical properties diverge markedly: ABW-Ge_4_ has a single nodal ring, whereas I229-Ge_48_ emerges as a topological semimetal with a sophisticated, nested nodal ring structure. Furthermore, under the influence of spin-orbit coupling (SOC), unlike ABW-Ge_4_, the nodal points in I229-Ge_48_ do not induce a substantial band gap opening, thereby preserving the stability of its topological semimetal characteristics under SOC.

### 3.5. Strain Control

**Figure 7:**
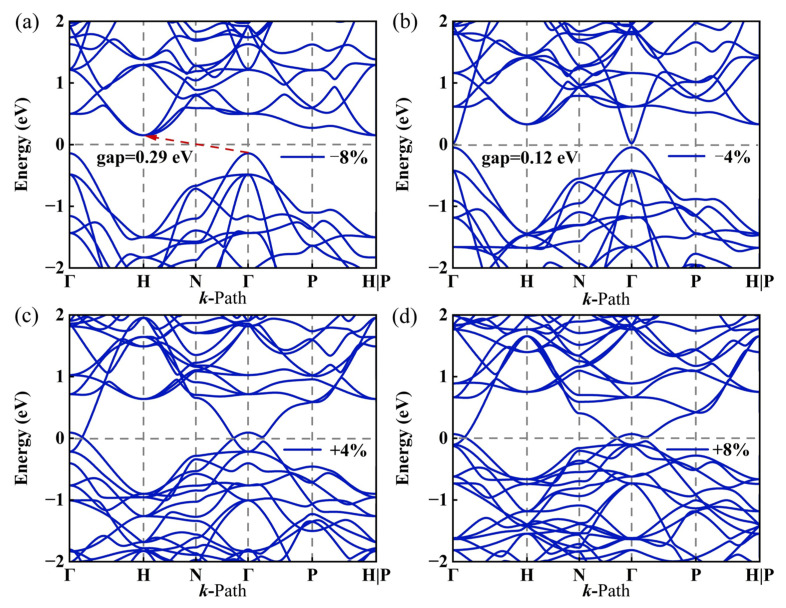
Calculated 2D energy band structure under triaxial strain at (**a**) ε = −8%, (**b**) ε = −4%, (**c**) ε = 4% and (**d**) ε = 8%.

Strain control of material properties is a promising method, so we study the change in energy band structure under triaxial strain ranging from −8% to 8%. As shown in Figure 7, the energy band of the I229-Ge_48_ undergoes significant changes under the influence of strain. (i) When compressive strain is applied, I229-Ge_48_ transitions from a semimetal to a direct bandgap semiconductor with a bandgap of 0.12 eV at −4% strain. Further increasing the compressive strain to −8%, it becomes an indirect bandgap semiconductor with a bandgap of 0.29 eV. (ii) When tensile strain is applied, the two energy band intersections near the Fermi level remain closed throughout the entire tensile process, indicating that the topological properties of I229-Ge_48_ do not change during the stretching process. This demonstrates that strain engineering plays a significant role in modulating the band structure.

### 3.6. Optical Properties

Exploration of the optical properties of I229-Ge_48_ offers insight into how this material interacts with light, which is essential for its potential applications in microelectronic and optoelectronic devices. The optical properties are characterized by the dielectric function $\varepsilon \left(\omega \right)=\varepsilon_{1}\left(\omega \right)+i\varepsilon_{2}\left(\omega \right)$, where $\varepsilon_{1}\left(\omega \right)$ represents the real part of the dielectric function, $\varepsilon_{2}\left(\omega \right)$ denotes the imaginary part, and ω symbolizes the frequency. The optical absorption coefficient of a material can be expressed as [73]

(7) $$\alpha (\omega )=\sqrt{2}\omega {\left[\sqrt{\varepsilon_{1}^{2}(\omega )+\varepsilon_{2}^{2}(\omega )}-\varepsilon_{1}(\omega )\right]}^{1/2}$$

Given that strain engineering can alter the electronic properties of materials, our discussion on the optical properties of intrinsic I229-Ge_48_ is complemented by an examination of the effects of strain. The optical absorption coefficient serves as a critical indicator of a material’s optical characteristics. Meanwhile, the imaginary part of the dielectric function is also correlated with the degree of optical absorption. Consequently, we investigated the optical absorption characteristic and how the imaginary part of the dielectric function varies with photon energy for I229-Ge_48_ under strain modulation.

**Figure 8:**
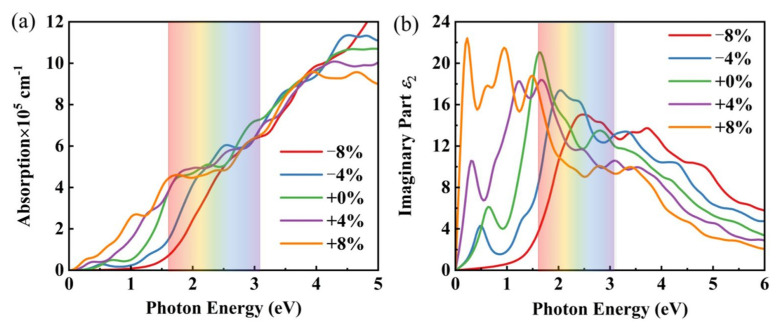
(**a**) Absorption coefficient of I229-Ge_48_ under different strains. (**b**) Imaginary part of the dielectric function of I229-Ge_48_ under different strains.

Analysis of the absorption spectra (Figure 8a) reveals that, in its unstrained state, I229-Ge_48_ exhibits light absorption coefficients within the visible spectrum reaching up to 10^5^ in magnitude, rivaling those of graphene and highlighting its exceptional light absorption capabilities. This broad absorption extends beyond the visible range, encompassing the near-infrared and ultraviolet regions, thereby positioning I229-Ge_48_ as a promising candidate for applications in solar photovoltaic cells and optoelectronic devices. Furthermore, introduction of 4% compressive strain induces an absorption peak within the visible light spectrum, augmenting the material’s light absorption efficiency. Conversely, subjecting the material to 8% tensile strain significantly enhances its absorption in the infrared range.

The imaginary part of the dielectric function reflects the light absorption capacity of a material. Figure 8b illustrates that I229-Ge_48_ exhibits a pronounced dielectric peak within the visible spectrum, accompanied by its initial dielectric peak emerging in the infrared band, signifying augmented light absorption across these spectral ranges. Notably, application of strain within the range of −8% to 8% induces a substantial redshift in the absorption curve, extending it into the infrared region. This shift underscores the material’s promise for integration into infrared sensing technologies. These findings are in accordance with the optical absorption coefficient, reinforcing the notion that I229-Ge_48_ possesses considerable scientific significance in the realm of optics. Moreover, they highlight the efficacy of strain manipulation as a versatile tool for tuning the optical characteristics of I229-Ge_48_, thereby broadening its potential utility in advanced photonic and optoelectronic devices.

## 4. Conclusions

To summarize, we have successfully predicted I229-Ge_48_, a novel cluster-assembled Ge allotrope. Our findings reveal several key characteristics. (I) I229-Ge_48_ is identified as a low-density nanoporous material, distinguished by its unique crystal structure. (II) I229-Ge_48_ has an anisotropic Young’s modulus and Poisson’s ratio distinct from other brittle Ge allotropes. (III) I229-Ge_48_ is a prominent topological semimetal characterized by a porous spherical Fermi surface, rendering it a compelling candidate for future studies to unravel novel quantum phenomena. (IV) Under the influence of SOC and tensile strain, I229-Ge_48_ is classified as a robust topological nodal line semimetal. Notably, applying compressive strain induces transition to a semiconducting state, highlighting the significant tunability of its energy band structure through strain engineering. (V) I229-Ge_48_ shows strong light absorption in the visible spectrum, and its optical properties are significantly altered by strain modulation, making it an attractive candidate for optoelectronic devices. This work advances the design of cluster-assembled topological materials and provides a platform for exploring strain-driven quantum phenomena in germanium-based systems.

## Figures and Tables

**Table 1:** Calculated elastic constants *C*_ij_ (GPa), bulk modulus *B* (GPa), shear modulus *G* (GPa), Young’s modulus *Y* (GPa), *B*/*G* ratio, Poisson’s ratio (*ν*), and universal anisotropy index *A*^U^ of I229-Ge_48_ and some other germanium structures at zero pressure.

Structure		*C*_11_	*C*_12_	*C*_13_	*C*_22_	*C*_23_	*C*_33_	*C*_44_	*C*_55_	*C*_66_	*B*	*G*	*B*/*G*	*Y*	*ν*	*A* ^u^
I229-Ge_48_	This work	42	26					20			31	14	2.21	37	0.30	0.770
Diamond	This work	116	45					61			69	50	1.38	121	0.21	0.313
	Cal. [59]	121	49					62			73	50	1.46	122	0.22	0.342
	Exp. [60]	129	48					67			77					
Ge_12_	This work	83	28	29			91	35		40	48	33	1.44	81	0.22	0.104
	Cal. [59]	88	26	32			100	35		40	50	34	1.47	83	0.22	0.054
oC24	This work	124	29	23	108	32	110	30	38	41	57	38	1.50	93	0.23	0.119
	Cal. [61]	124	32	20	107	30	118	29	38	40	57	39	1.46	95	0.22	
Ge_20_	This work	112	33	37			98	50		40	60	42	1.43	102	0.22	0.140
	Cal. [62]	112	32	37			96	49		39	59	41	1.44	100	0.22	0.153
ST12	This work	139	18	24			77	45		36	54	44	1.23	104	0.18	0.358
	Exp. [63]										55	42				
hcp	This work	94	39	47			65	26		27	57	23	2.53	60	0.33	0.591

## Data Availability

The data that support the plots within this paper and other findings of this study are available from the corresponding author upon reasonable request.

## Supplementary Materials

The following supporting information can be downloaded at: https://www.mdpi.com/article/10.3390/nano15141109/s1, Table S1: The space group (SG), lattice constants a, b, and c (in Å), volume V (in Å3/atom), equilibrium density ρ (in g/cm3), total energy Etot (in eV/atom), and energy difference between the diamond structure and the other structure ∆E (in eV/atom) for I229-Ge48, Diamond, Ge12, oC24, Ge20, ST12, and hcp phase. Figure S1: The energy band structures of I229-Ge48 calculated via the modified Beck–Johnson exchange potential. Figure S2: Fermi surface of I229-Ge_48_ with SOC action.

## References

[1] Wang Z., Weng H., Wu Q., Dai X., Fang Z. (2013). Three-Dimensional Dirac Semimetal and Quantum Transport in Cd_3_As_2_. Phys. Rev. B.

[2] Liu Z.K., Zhou B., Zhang Y., Wang Z.J., Weng H.M., Prabhakaran D., Mo S.-K., Shen Z.X., Fang Z., Dai X. (2014). Discovery of a Three-Dimensional Topological Dirac Semimetal, Na_3_Bi. Science.

[3] Hosur P. (2012). Friedel Oscillations Due to Fermi Arcs in Weyl Semimetals. Phys. Rev. B.

[4] Bian G., Chang T.-R., Sankar R., Xu S.-Y., Zheng H., Neupert T., Chiu C.-K., Huang S.-M., Chang G., Belopolski I. (2016). Topological Nodal-Line Fermions in Spin-Orbit Metal PbTaSe_2_. Nat. Commun..

[5] Cheng Y., Liu D., Liu X., Zhang R., Cui X., Liu Z., Song T. (2024). High-Throughput Screening for Ideal 3D Carbon Topological Semimetals via Bottom-up Approach. J. Phys. Chem. C.

[6] Wang L., Zhao M., Wang J., Liu Y., Liu G., Wang X., Zhang G., Zhang X. (2023). High-Performance Hydrogen Evolution Reaction Catalysts in Two-Dimensional Nodal Line Semimetals. ACS Appl. Mater. Interfaces.

[7] Kong X., Liu Z., Geng Z., Zhang A., Guo Z., Cui S., Xia C., Tan S., Zhou S., Wang Z. (2024). Experimental Demonstration of Topological Catalysis for CO_2_ Electroreduction. J. Am. Chem. Soc..

[8] Nishihaya S., Uchida M., Nakazawa Y., Kurihara R., Akiba K., Kriener M., Miyake A., Taguchi Y., Tokunaga M., Kawasaki M. (2019). Quantized Surface Transport in Topological Dirac Semimetal Films. Nat. Commun..

[9] Son D.T., Spivak B.Z. (2013). Chiral Anomaly and Classical Negative Magnetoresistance of Weyl Metals. Phys. Rev. B.

[10] Huang X., Zhao L., Long Y., Wang P., Chen D., Yang Z., Liang H., Xue M., Weng H., Fang Z. (2015). Observation of the Chiral-Anomaly-Induced Negative Magnetoresistance in 3D Weyl Semimetal TaAs. Phys. Rev. X.

[11] Gao H., Venderbos J.W.F., Kim Y., Rappe A.M. (2019). Topological Semimetals from First Principles. Annu. Rev. Mater. Res..

[12] Cao W., Tang P., Zhang S.-C., Duan W., Rubio A. (2016). Stable Dirac Semimetal in the Allotropes of Group-IV Elements. Phys. Rev. B.

[13] Wang Z., Sun Y., Chen X.-Q., Franchini C., Xu G., Weng H., Dai X., Fang Z. (2012). Dirac Semimetal and Topological Phase Transitions in A_3_Bi (A = Na, K, Rb). Phys. Rev. B.

[14] Wang Z., Liu D., Teo H.T., Wang Q., Xue H., Zhang B. (2022). Higher-Order Dirac Semimetal in a Photonic Crystal. Phys. Rev. B.

[15] Wan X., Turner A.M., Vishwanath A., Savrasov S.Y. (2011). Topological Semimetal and Fermi-Arc Surface States in the Electronic Structure of Pyrochlore Iridates. Phys. Rev. B.

[16] Xu G., Weng H., Wang Z., Dai X., Fang Z. (2011). Chern Semimetal and the Quantized Anomalous Hall Effect in HgCr_2_Se_4_. Phys. Rev. Lett..

[17] Li X.-P., Deng K., Fu B., Li Y., Ma D.-S., Han J., Zhou J., Zhou S., Yao Y. (2021). Type-III Weyl Semimetals: (TaSe_4_)_2_I. Phys. Rev. B.

[18] Hořava P. (2005). Stability of Fermi Surfaces and K Theory. Phys. Rev. Lett..

[19] Burkov A.A., Hook M.D., Balents L. (2011). Topological Nodal Semimetals. Phys. Rev. B.

[20] Abramovich S., Dutta D., Rizza C., Santoro S., Aquino M., Cupolillo A., Occhiuzzi J., Russa M.F.L., Ghosh B., Farias D. (2022). NiSe and CoSe Topological Nodal-Line Semimetals: A Sustainable Platform for Efficient Thermoplasmonics and Solar-Driven Photothermal Membrane Distillation. Small.

[21] Novoselov K.S., Geim A.K., Morozov S.V., Jiang D., Zhang Y., Dubonos S.V., Grigorieva I.V., Firsov A.A. (2004). Electric Field Effect in Atomically Thin Carbon Films. Science.

[22] Vogt P., De Padova P., Quaresima C., Avila J., Frantzeskakis E., Asensio M.C., Resta A., Ealet B., Le Lay G. (2012). Silicene: Compelling Experimental Evidence for Graphenelike Two-Dimensional Silicon. Phys. Rev. Lett..

[23] Li L., Lu S., Pan J., Qin Z., Wang Y., Wang Y., Cao G., Du S., Gao H. (2014). Buckled Germanene Formation on Pt(111). Adv. Mater..

[24] Chen Y., Xie Y., Yang S.A., Pan H., Zhang F., Cohen M.L., Zhang S. (2015). Nanostructured Carbon Allotropes with Weyl-like Loops and Points. Nano Lett..

[25] Zhong C., Chen Y., Xie Y., Yang S.A., Cohen M.L., Zhang S.B. (2016). Towards Three-Dimensional Weyl-Surface Semimetals in Graphene Networks. Nanoscale.

[26] Zhong C., Xie Y., Chen Y., Zhang S. (2016). Coexistence of Flat Bands and Dirac Bands in a Carbon-Kagome-Lattice Family. Carbon.

[27] Qie Y., Liu J., Li X., Wang S., Sun Q., Jena P. (2018). Interpenetrating Silicene Networks: A Topological Nodal-Line Semimetal with Potential as an Anode Material for Sodium Ion Batteries. Phys. Rev. Mater..

[28] Kasper E., Oehme M., Lupaca-Schomber J. (2008). High Ge Content SiGe Alloys: Doping and Contact Formation. ECS Trans..

[29] Barkalov O.I., Tissen V.G., McMillan P.F., Wilson M., Sella A., Nefedova M.V. (2010). Pressure-Induced Transformations and Superconductivity of Amorphous Germanium. Phys. Rev. B.

[30] Pillarisetty R. (2011). Academic and Industry Research Progress in Germanium Nanodevices. Nature.

[31] Menoni C.S., Hu J.Z., Spain I.L. (1986). Germanium at High Pressures. Phys. Rev. B.

[32] Tanaka K. (1991). Amorphous Ge under Pressure. Phys. Rev. B.

[33] Bundy F.P., Kasper J.S. (1963). A New Dense Form of Solid Germanium. Science.

[34] Arguilla M.Q., Jiang S., Chitara B., Goldberger J.E. (2014). Synthesis and Stability of Two-Dimensional Ge/Sn Graphane Alloys. Chem. Mater..

[35] Nguyen M.C., Zhao X., Wang C.-Z., Ho K.-M. (2014). *Sp*^3^-Hybridized Framework Structure of Group-14 Elements Discovered by Genetic Algorithm. Phys. Rev. B.

[36] Lan H.-S., Chang S.T., Liu C.W. (2017). Semiconductor, Topological Semimetal, Indirect Semimetal, and Topological Dirac Semimetal Phases of Ge_1−x_Sn_x_ Alloys. Phys. Rev. B.

[37] Zou Y., Wu N., Song T., Liu Z., Cui X. (2023). From Topological Nodal-Line Semimetal to Insulator in ABW-Ge_4_: A New Member of the Germanium Allotrope. ACS Omega.

[38] Zhu Z., Li M., Li J. (2016). Topological Semimetal to Insulator Quantum Phase Transition in the Zintl Compounds Ba_2_X (X = Si, Ge). Phys. Rev. B.

[39] Bampoulis P., Castenmiller C., Klaassen D.J., van Mil J., Liu Y., Liu C.-C., Yao Y., Ezawa M., Rudenko A.N., Zandvliet H.J.W. (2023). Quantum Spin Hall States and Topological Phase Transition in Germanene. Phys. Rev. Lett..

[40] Basnet R., Upreti D., McCarthy T.T., Ju Z., McMinn A.M., Sharma M.M., Zhang Y.-H., Hu J. (2024). Magneto-Transport Study on Sn-Rich Sn_1−x_Ge_x_ Thin Films Enabled by CdTe Buffer Layer. J. Vac. Sci. Technol. B.

[41] Claridge S.A., Castleman A.W., Khanna S.N., Murray C.B., Sen A., Weiss P.S. (2009). Cluster-Assembled Materials. ACS Nano.

[42] Kresse G., Furthmüller J. (1996). Efficiency of Ab-Initio Total Energy Calculations for Metals and Semiconductors Using a Plane-Wave Basis Set. Comput. Mater. Sci..

[43] Blöchl P.E. (1994). Projector Augmented-Wave Method. Phys. Rev. B.

[44] Kresse G., Furthmüller J. (1996). Efficient Iterative Schemes for Ab Initio Total-Energy Calculations Using a Plane-Wave Basis Set. Phys. Rev. B.

[45] Perdew J.P., Wang Y. (1992). Accurate and Simple Analytic Representation of the Electron-Gas Correlation Energy. Phys. Rev. B.

[46] Perdew J.P., Burke K., Ernzerhof M. (1996). Generalized Gradient Approximation Made Simple. Phys. Rev. Lett..

[47] Kohn W., Sham L.J. (1965). Self-Consistent Equations Including Exchange and Correlation Effects. Phys. Rev..

[48] Togo A., Tanaka I. (2015). First Principles Phonon Calculations in Materials Science. Scr. Mater..

[49] Martyna G.J., Klein M.L., Tuckerman M. (1992). Nosé–Hoover Chains: The Canonical Ensemble via Continuous Dynamics. J. Chem. Phys..

[50] Gao J., Wu Q., Persson C., Wang Z. (2021). Irvsp: To Obtain Irreducible Representations of Electronic States in the VASP. Comput. Phys. Commun..

[51] Mostofi A.A., Yates J.R., Lee Y.-S., Souza I., Vanderbilt D., Marzari N. (2008). Wannier90: A Tool for Obtaining Maximally-Localised Wannier Functions. Comput. Phys. Commun..

[52] Wu Q., Zhang S., Song H.-F., Troyer M., Soluyanov A.A. (2018). WannierTools: An Open-Source Software Package for Novel Topological Materials. Comput. Phys. Commun..

[53] Oku T., Nishiwaki A., Narita I., Gonda M. (2003). Formation and Structure of B_24_N_24_ Clusters. Chem. Phys. Lett..

[54] Lide D.R. (1994). CRC Handbook of Chemistry and Physics.

[55] Kim E.H., Shin Y.-H., Lee B.-J. (2008). A Modified Embedded-Atom Method Interatomic Potential for Germanium. Calphad.

[56] Birch F. (1947). Finite Elastic Strain of Cubic Crystals. Phys. Rev..

[57] Silvi B., Savin A. (1994). Classification of Chemical Bonds Based on Topological Analysis of Electron Localization Functions. Nature.

[58] Hill R. (1952). The Elastic Behaviour of a Crystalline Aggregate. Proc. Phys. Soc. A.

[59] Fan Q., Chai C., Wei Q., Yang Q., Zhou P., Xing M., Yang Y. (2016). Mechanical and Electronic Properties of Si, Ge and Their Alloys in *P*4_2_/*Mnm* Structure. Mater. Sci. Semicond. Process..

[60] Gómez-Abal R., Li X., Scheffler M., Ambrosch-Draxl C. (2008). Influence of the Core-Valence Interaction and of the Pseudopotential Approximation on the Electron Self-Energy in Semiconductors. Phys. Rev. Lett..

[61] Fan Q., Sun Y., Zhao Y., Song Y., Yun S. (2023). Group 14 Elements in the *Cmcm* Phase with a Direct Band Structure for Photoelectric Application. Phys. Scr..

[62] Fan Q., Hao B., Jiang L., Yu X., Zhang W., Song Y., Yun S. (2021). Group 14 Semiconductor Alloys in the *P*4_1_2_1_2 Phase: A Comprehensive Study. Results Phys..

[63] Yuan Q., Li S., Zhou L., He D. (2022). Phase-Pure ST12 Ge Bulks through Secondary Pressure Induced Phase Transition. Solid State Commun..

[64] Pugh S.F. (1954). XCII. Relations between the Elastic Moduli and the Plastic Properties of Polycrystalline Pure Metals. Philos. Mag..

[65] Nye J.F. (1985). Physical Properties of Crystals.

[66] Ranganathan S.I., Ostoja-Starzewski M. (2008). Universal Elastic Anisotropy Index. Phys. Rev. Lett..

[67] Tran F., Blaha P. (2009). Accurate Band Gaps of Semiconductors and Insulators with a Semilocal Exchange-Correlation Potential. Phys. Rev. Lett..

[68] Soluyanov A.A., Gresch D., Wang Z., Wu Q., Troyer M., Dai X., Bernevig B.A. (2015). Type-II Weyl Semimetals. Nature.

[69] Sharma M.M., Chhetri S.K., Acharya G., Graf D., Upreti D., Dahal S., Nabi M.R.U., Rahman S., Sakon J., Churchill H.O.H. (2025). Quantum Oscillation Studies of the Nodal Line Semimetal Ni_3_In_2_S_2−x_Se_x_. Acta Mater..

[70] Meng J., Liu G., Zhang W., Zhao L., Liu H., Jia X., Mu D., Liu S., Dong X., Zhang J. (2009). Coexistence of Fermi Arcs and Fermi Pockets in a High-Tc Copper Oxide Superconductor. Nature.

[71] Damascelli A., Hussain Z., Shen Z.-X. (2003). Angle-Resolved Photoemission Studies of the Cuprate Superconductors. Rev. Mod. Phys..

[72] Sharma M.M., Karn N.K., Rani P., Bhowmik R.N., Awana V.P.S. (2022). Bulk Superconductivity and Non-Trivial Band Topology Analysis of Pb_2_Pd. Supercond. Sci. Technol..

[73] Cui Y., Peng L., Sun L., Qian Q., Huang Y. (2018). Two-Dimensional Few-Layer Group-III Metal Monochalcogenides as Effective Photocatalysts for Overall Water Splitting in the Visible Range. J. Mater. Chem. A.

