# Supplementary material for: First-Principles Study of Topological Nodal Line Semimetal I229-Ge48 via Cluster Assembly
Source: Nanomaterials (Basel). 2025 Jul 17;15(14):1109. doi: 10.3390/nano15141109 (PMC12298778; doi:10.3390/nano15141109)
Supplement: Supplementary file 1 [file nanomaterials-15-01109-s001.zip › nanomaterials-3727579-supplementary.pdf]

nanomaterials

Article

First-principles study of topological nodal line semimetal I229-Ge_{48} via cluster assembly

Liwei Liu, Xin Wang, Nan Wang, Yaru Chen, Shumin Wang, Caizhi Hua, Tielei Song, Zhifeng Liu and Xin Cui *

Inner Mongolia Key Laboratory of Microscale Physics and Atom Innovation, School of Physical Science and Technology, Inner Mongolia University, Hohhot 010021, China

* Correspondence: pycuixin@imu.edu.cn; Tel.: +8613204711862

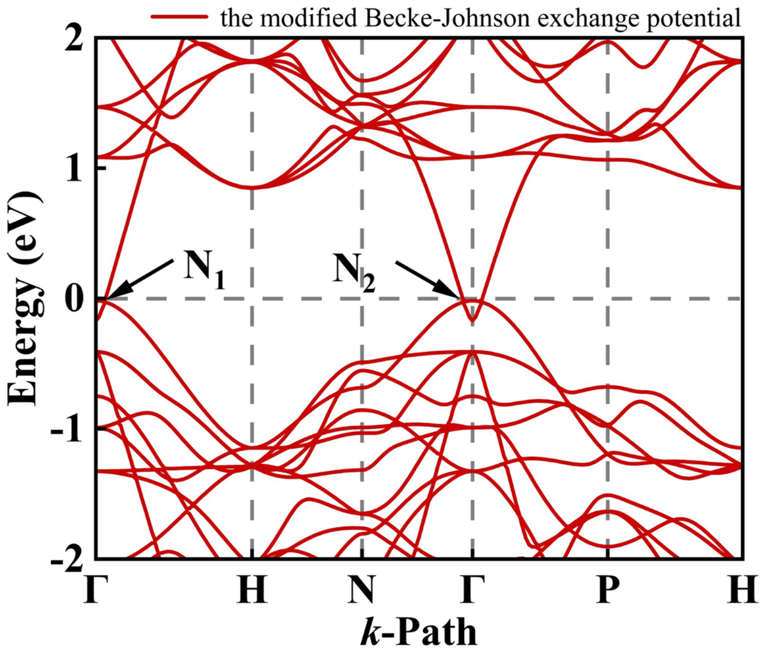

Figure S1. The energy band structures of I229-Ge_{48} calculated via the modified Becke-Johnson exchange potential.

Academic Editor: Firstname Last-name

Received: date
Revised: date
Accepted: date
Published: date

Citation: To be added by editorial staff during production.

Copyright: © 2025 by the authors. Submitted for possible open access publication under the terms and conditions of the Creative Commons Attribution (CC BY) license (https://creativecommons.org/licenses/by/4.0/).

Table S1. The space group (*SG*), lattice constants *a*, *b* and *c* (in Å), volume *V* (in Å^3/atom), equilibrium density *ρ* (in g/cm^3), total energy E_{tot} (in eV/atom) and the energy difference between the Diamond structure and the other structure *ΔE* (in eV/atom) for I229-Ge_{48}, Diamond, Ge_{12}, oC24, Ge_{20}, ST12 and hcp phases.

Structure	*SG*		*a* (Å)	*b* (Å)	*c* (Å)	*V* (Å^3)	*ρ* (g/cm^3)	E_{tot} (eV)	*ΔE* (eV/atom))
I229-Ge_{48}	*Im*-3*m*	This work	12.199			1808.527	3.201	–4.201	0.312
Diamond	*Fd*-3*m*	This work	5.694			184.609	5.226	–4.513	0.000
		Cal.[59]	5.694				5.224		
		Exp.[54]	5.660				5.318		
Ge_{12}	*P*4$_2$/*mnm*	This work	5.649		10.150	323.888	4.468	–4.432	0.081
		Cal.[59]	5.638		10.149				
oC24	*Cmcm*	This work	4.081	21.162	6.882	594.283	4.871	–4.452	0.061
		Cal.[61]	4.057	21.114	6.862		4.922		
Ge_{20}	*P*4$_1$2$_1$2	This work	9.368		5.525	484.804	4.975	–4.481	0.032
		Cal.[62]	9.338		5.508		5.019		
ST12	*P*4$_3$2$_1$2	This work	6.012		7.156			–4.385	0.128
		Exp.[63]	5.930		6.979	245.450			
hcp	*P*6$_3$/*mmc*	This work	3.014		4.894	38.488	6.267	–4.174	0.339
		Cal.[55]	2.960		4.825				

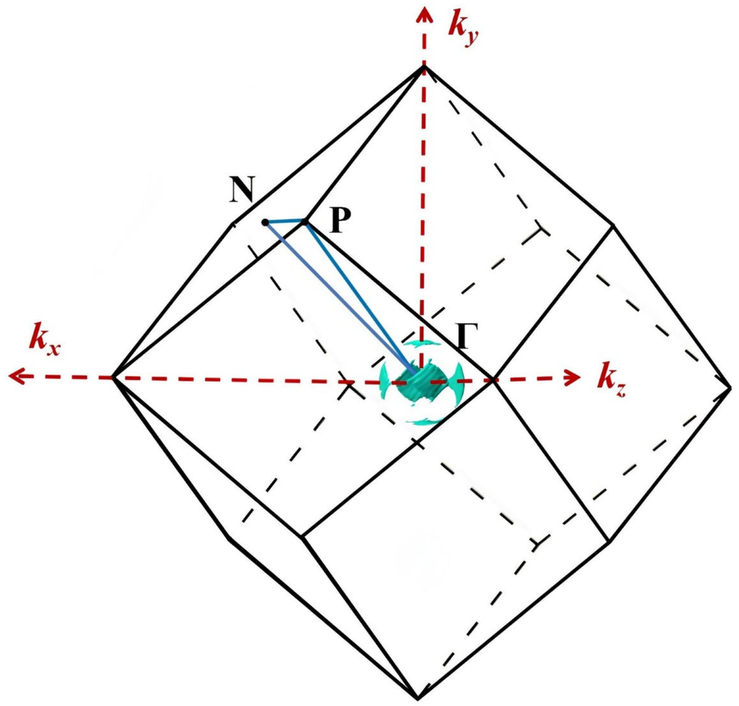

Figure S2. Fermi surface of I229-Ge_{48} with SOC action.

References

54. Lide, D. R. *CRC Handbook of Chemistry and Physics*, 73rd ed.; CRC Press: Boca Raton, FL, USA, **1994.**
55. Kim, E.H.; Shin, Y.-H.; Lee, B.-J. A Modified Embedded-Atom Method Interatomic Potential for Germanium. *Calphad* **2008**, *32*, 34–42.
59. Fan, Q.; Chai, C.; Wei, Q.; Yang, Q.; Zhou, P.; Xing, M.; Yang, Y. Mechanical and Electronic Properties of Si, Ge and Their Alloys in $P4_2/Mnm$ Structure. *Mater. Sci. Semicond. Process.* **2016**, *43*, 187–195.
61. Fan, Q.; Sun, Y.; Zhao, Y.; Song, Y.; Yun, S. Group 14 Elements in the *Cmcm* Phase with a Direct Band Structure for Photoelectric Application. *Phys. Scr.* **2023**, *98*, 015701.
62. Fan, Q.; Hao, B.; Jiang, L.; Yu, X.; Zhang, W.; Song, Y.; Yun, S. Group 14 Semiconductor Alloys in the $P4_12_12$ Phase: A Comprehensive Study. *Results Phys.* **2021**, *25*, 104254.
63. Yuan, Q.; Li, S.; Zhou, L.; He, D. Phase-Pure ST12 Ge Bulks through Secondary Pressure Induced Phase Transition. *Solid State Commun.* **2022**, *348–349*, 114742.
